# The Antioxidant Polysaccharide from *Semiaquilegia adoxoides* (DC.) Makino Adjusts the Immune Response of Mice Infected by Bacteria

**DOI:** 10.1155/2020/2719483

**Published:** 2020-02-19

**Authors:** Yunqiao Yang, Lei Guo, Kinza Tariq, Weiyu Zhang, Chong Li, Fareed Qamar Memon, Beibei Chai, Zheng Li, Junying Sun, Yunru Chen, Geyin Zhang, Qinmei Li, Shuaiyang Wang, Lizhen Wang, Chongbo Lai, Mingsheng Jiang, Hongbin Si

**Affiliations:** ^1^College of Animal Sciences and Technology, Guangxi University, Nanning, China; ^2^College of Bioscience and Biotechnology, Yangzhou University, Yangzhou, China

## Abstract

*Semiaquilegia adoxoides* (DC.) Makino is a herbal medicine and it is recorded that its water extract can be used to treat acute diseases caused by bacterial infections. In order to understand the polysaccharide of *Semiaquilegia adoxoides* (DC.) Makino (SMP), FT-IR and HPLC methods were performed to determine the basic chemical structure and monosaccharide compositions of SMP. The antioxidant capacity of SMP was analyzed by monitoring both the scavenging rate of DPPH and ABTS free radical. To investigate the effects of SMP on the acute bacterial disease, minimum inhibitory concentrations (MICs) of SMP on *E. coli* or *S. aureus* were detected; meanwhile, mice were administrated with SMP for 7 days and then infected with *E. coli* or *S. aureus*, and the parameters were measured at the 9^th^ day. Results showed that SMP was a furanose which was mainly composed of glucose (60.3%) and had certain antioxidant activities. Both MIC values of SMP on *E. coli* and *S. aureus* were 250 ml/mL, which means that SMP has no direct antibacterial effects. The mice experiments revealed that SMP had potential effects on immunomodulatory by reducing WBC and the expression of serum IL-1, IL-6, and TNF-*α* and increasing IgM of *E. coli* or *S. aureus* infected mice. These findings supported the effect of *Semiaquilegia adoxoides* (DC.) Makino in folk use with scientific evidence.

## 1. Introduction

Our body is surrounded by microbes, including *Escherichia coli* (*E. coli*), *Staphylococcus aureus* (*S. aureus*), and other pathogenic microorganisms. These bacteria can cause nosocomial and farm infection which can result in serious infections [1]. In recent years, the abuse of antibiotics in the treatment of bacterial diseases has led to the emergence of a variety of antibiotic-resistant bacteria, and the search for alternatives has become an urgent need. Immune-related cells block the spread and infection of pathogenic bacteria, but the components which are released by bacteria can also influence the immune system. Therefore, the use of special compounds to kill bacteria and/or enhance the body's immune system can help to deal with the infection by bacteria [2].

*Semiaquilegia adoxoides* (DC.) Makino (Ranunculaceae) *(S. Makino)* is a medicinal plant and it is the only member of *Semiaquilegia* genus which distribute in Japan and Subtropical Region of the Yangtze River Basin in China [3]. Pharmacological research indicated that the n-butanol extract of *S. Makino* protects human lens epithelial cells in oxidative stress which was induced by hydrogen peroxide [4], and the ethanol extraction of *S. Makino* has certain anticancer activity [5]. According to the documentation in ancient China, the powers or aqueous extractions of *S. Makino* have been widely used in the treatment of furuncle swelling, tonsillitis, and mammary abscess which are often associated with *S. aureus* and *E. coli* in clinical infection according to modern medical theory [5, 6]. Plant polysaccharides are soluble in water and have a relatively high content in water extraction, and the multiple biological functions of polysaccharides from nature plants were well recorded in recent literatures, such as antibacterial [7], antioxidant [8], antitumor [9], and immune activities [10]. However, few reports covered the basic structure, antioxidant, and biological activities of polysaccharides of *S. Makino*.

The purposes of this work were to evaluate (1) the antioxidative effects of *S. Makino* polysaccharides (SMP) by DPPH and ABTS methods, (2) the inhibitory effect of SMP on the growth of *S. aureus* or *E. coli* by serial-dilution culture method, and (3) the immunomodulatory activity of SMP on *S. aureus* or *E. coli* infected mice by mortality and cytokine production in serum. Additionally, the monosaccharide compositions and Fourier-transform infrared spectroscopy (FT-IR) analysis of SMP were also conducted.

## 2. Materials and Methods

### 2.1. Materials and Reagents

Standard monosaccharide samples were purchased from Sigma Chemicals Company (St. Louis, USA). ELISA kits for assaying the IL-1, IL-6, TNF-*α*, and IgM in serum were purchased from Shanghai E-research Company (Shanghai, China). Amikacin and vancomycin were supplied by our laboratory. All other reagents and chemicals were of analytical grade and purchased from local chemical suppliers.

### 2.2. Extraction

*Semiaquilegia adoxoides* (DC.) Makino (Ranunculaceae) (500 g) were grounded into powder and macerated with 1000 ml anhydrous ethanol overnight for the removal of lipids. The precipitates were collected and heated with 2000 ml double-distilled water (ddH_2_O) at 85°C for 3 h for 3 times. All the extractions of *S. Makino* were filtered and then centrifuged at 9000 rpm for 5 min. The supernatants were combined and concentrated at 85°C to 500 ml and precipitated with three volumes of anhydrous ethanol (75%, v/v) overnight at 4°C and then centrifuged again as above. The precipitate was dissolved in ddH_2_O and then after 7 times of deproteinization by Sevag method [11], the precipitation process was repeated with anhydrous ethanol (95%, v/v) three times. The final precipitate was washed consecutively with acetone and ether and then freeze-dried to obtain the SMP. The content of polysaccharides in *S. Makino* was determined by phenol sulphuric acid assay [12].

### 2.3. Fourier-Transform Infrared Spectroscopy (FT-IR) Analysis

2.0 mg SMP sample was mixed with 200 mg KBr. After grinding and pressing into KBr pellet, scanning was performed between the ranges of 4000 cm^−1^ to 400 cm^−1^ with a resolution of 4 cm^−1^.

### 2.4. Analysis of SMP Monosaccharide Composition

The monosaccharide composition of SMP was detected by high-performance liquid chromatography (HPLC) after acid hydrolysis according to the report [13]. Briefly, the SMP sample (10 mg) was hydrolyzed with trifluoroacetic acid (TFA, 2 M, 3 mL) at 121°C for 2 h in a tube. Residual TFA was removed with a QGC-12T nitrogen blowing instrument at 50°C. The hydrolyzed samples were then redissolved in ddH_2_O and analyzed with ICS 5000 ion chromatography (Dionex, Sunnyvale, CA) with a CarboPac PA20 analytic column (150 mm × 3 mm inner diameter) and a pulsed amperometric detector. The mobile phase consisted of 250 mM NaOH (2%) and water (98%) at a flow rate of 0.5 mL·min^−1^.

### 2.5. DPPH Radical Scavenging Assay

3.0 ml sample solution with various concentrations (0.3–5 mg/mL) was mixed with 2.0 ml DPPH solution (0.2 mM, dissolved in ethanol). After being incubated for 30 min in the dark at 25°C, the absorbance of the mixture against blank was determined at 520 nm. The DPPH radical scavenging activity was calculated as the percentage of inhibition according to the following equation:

(1) $$\begin{array}{c} =\left(1-\frac{A_{\text{s}}-A_{\text{s}0}}{A_{0}}\right)\times 100\%. \end{array}$$

In this equation, *A*_0_ is the absorbance of a blank treatment group, *A*_s_ is the absorbance of a sample treatment group, and *A*_s0_ is the absorbance of a sample background. All measurements were performed in triplicate and ascorbic acid (Vitamin C) was used as a positive control.

### 2.6. ABTS Radical Scavenging Assay

The ABTS^•+^ was prepared by mixing an ABTS stock solution (7 mM in water) with 2.45 mM potassium persulfate. This mixture was kept still for 16 h at 25°C in the dark. The ABTS^•+^ working solution was obtained by diluting the stock solution in methanol to an absorbance of 0.7 ± 0.10 at 747 nm. 0.5 ml diluted sample was added to 5.0 ml ABTS^•+^ working solution and mixed thoroughly. The reaction mixture was kept at 25°C in the dark for 6 min, and the absorbance was recorded at 747 nm. ABTS radical scavenging activity was calculated as follows:

(2) $$\begin{array}{c} =\left(1-\frac{A_{\text{s}}-A_{\text{s}0}}{A_{0}}\right)\times 100\%. \end{array}$$

In this equation, *A*_0_ is the absorbance of a blank treatment group, *A*_s_ is the absorbance of a sample treatment group, and *A*_s0_ is the absorbance of a sample background. All measurements were performed in triplicate and ascorbic acid (Vitamin C) was used as a positive control.

### 2.7. Animals and Bacterial Strains

Young adult males (average weight 18–22 g) and females (average weight 18–22 g) SPF mice were purchased from Guangxi Medical University Laboratory Animal Center. The mice were kept in well-ventilated cages in the animal houses of Guangxi University and provided with sterilized water and complete formula feed and housed in a rodent facility at 25°C with a 12 h light-dark cycle for acclimatization. All procedures involving animals and their care used were approved by the Ethics Committee of Guangxi University. Experiments were started after the mice acclimating for a week.

*E. coli* and *S. aureus* were identified and provided by our laboratory.

### 2.8. Determination of Minimum Inhibitory Concentrations (MICs)

MIC values of SMP against 2 bacterial species were determined by the serial-dilution culture method.

TSB was used as the incubation medium. The SMP solution was serially diluted by TSB. All tubes (13 × 100 mm) contained 1.75 ml SMP solution and 1.75 ml of diluted bacterial inoculum (approximately 10^4^ CFU/ml), with a final concentration of SMP solution of 0.49, 0.98, 1.95, 3.91, 7.81, 15.63, 31.25, 62.50, 125.00, 250.00, and 500.00 mg/ml. After incubation at 37°C for 24 h, MICs were measured by visual inspection of the turbidity of broth in tubes [14]. Amikacin was used as a positive control drug of anti-*E. coli* and vancomycin was used as a positive anti-*S. aureus* control. All assays were carried out in triplicate.

### 2.9. Experimental Design

168 mice were randomly divided into seven groups including the control group, *E. coli* group, *E. coli* + amikacin group, *E. coli* + SMP group, *S. aureus* group, *S. aureus* + vancomycin group, and *S. aureus* + SMP group. Each group contained 12 females and 12 males. Each mouse in the *E. coli* + SMP group and *S. aureus* + SMP group was administrated by gavage with 0.1 mL/10 g of SMP (1 g/mL) one time every day, while mice in the other groups were orally administered with the same volume of ddH_2_O. On the 7^th^ day after treatment with SMP, each mouse in the *E. coli* group, *E. coli* + amikacin group, and *E. coli* + SMP group was intraperitoneally injected with 0.10 mL/10 g of 2 × 10^8^ CFU/ml (LD50) of *E. coli*. At the same time, in the *S. aureus* group, *S. aureus* + vancomycin group, and *S. aureus* + SMP group, each mouse was intraperitoneally injected with 0.10 mL/10 g of 3.7 × 10^6^ CFU/ml (LD50) of *S. aureus*, while mice in the control group were intraperitoneally injected with the same volume of sterile PBS. Mice in the *E. coli* + amikacin group or *S. aureus* + vancomycin group were infected with *E. coli* or *S. aureus* and subcutaneously injected with amikacin (60 mg/kg) or vancomycin (50 mg/kg) at 5 hours after infection, respectively, which means that these groups were for positive drug controls. The number of deaths in each group of mice was recorded after mice were injected with bacteria. After 42 hours of infection, mice were starved for food and water for 6 hours before being executed. Blood and serum were collected in each animal. In other words, the polysaccharides treated group was administered with SMP for 9 days and all the trials lasted for 9 days.

### 2.10. White Blood Cell Count

Blood samples were collected into an EDTA tube for white blood cell (WBC) counts.

### 2.11. Analysis of Cytokine Production and Antibody in the Serum

Analysis of IL-1, IL-6, TNF-*α*, and IgM in serum samples was performed according to the commercially ELISA kit manual.

### 2.12. Statistical Analysis

Statistical analysis was performed using SPSS (version 16.0, SPSS Inc., Chicago, IL, USA). Differences between the mortality rates were analyzed using the chi-square test. For DPPH and ABTS radical scavenging activity rates, enumeration of WBC, and serum IL-1, IL-6, TNF-*α*, and IgM levels, differences between groups were analyzed using one-way analysis of variance (ANOVA) followed by post hoc analysis using Tukey test. Differences were considered significant at *P* < 0.05.

## 3. Results and Discussion

### 3.1. DPPH and ABTS Radical Scavenging Activity

**Figure 1:**
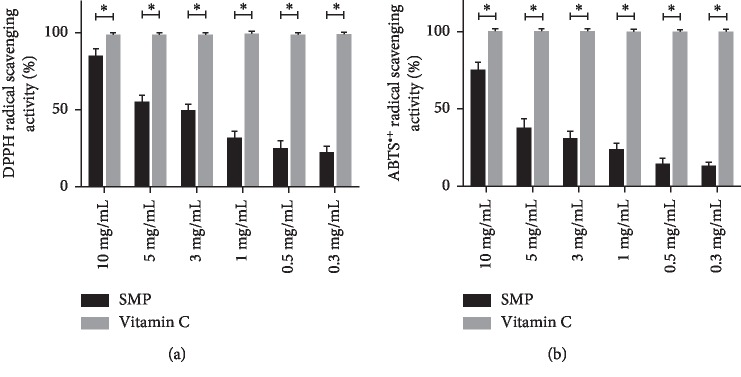
DPPH and ABTS radical scavenging activity of SMP. (a) DPPH radicals and (b) ABTS radicals. Note: ^*∗*^*P* < 0.05.

DPPH method is often used; meanwhile, it is an easy and rapid way to evaluate the free radical scavenging activity of polysaccharide and other natural compounds, as DPPH radicals are stable at room temperature but can be easily scavenged by antioxidants [15]. As shown in Figure 1(a), the SMP and positive control (Vitamin C) showed scavenged activities on DPPH radicals. 84.1% of DPPH scavenging efficiency was obtained at a concentration of 10 mg/ml of SMP, which showed a significant difference with that of Vitamin C (*P*=0.002). This DPPH scavenging activity was also correlated with the increasing concentrations of SMP.

The assay of ABTS radical cation (ABTS^+^) scavenging is also a commonly applied way to measure the antioxidant activity of natural products [16]. ABTS radical scavenging activities of SMP were determined and the results are shown in Figure 1(b). The highest scavenging ratios generated by 10 mg/ml of SMP were referred to be 74.8%, which was significantly lower than that of Vitamin C (*P*=0.003). The ABTS radical scavenging activities of SMP were also increased in a concentration-dependent manner and the positive control (Vitamin C) also had higher radical scavenging activities than that of SMP, which was over 97% at all concentrations and similar to the results of DPPH in Figure 1(a). This result also confirmed the positive correlation between DPPH and ABTS radical scavenging assay on antioxidant activity [17].

However, it is difficult to explain the scavenging mechanisms of polysaccharides on DPPH and ABTS radicals as the complexity of constituent and structure of this carbohydrate.

### 3.2. FT-IR Spectroscopy Analysis of SMP

**Figure 2:**
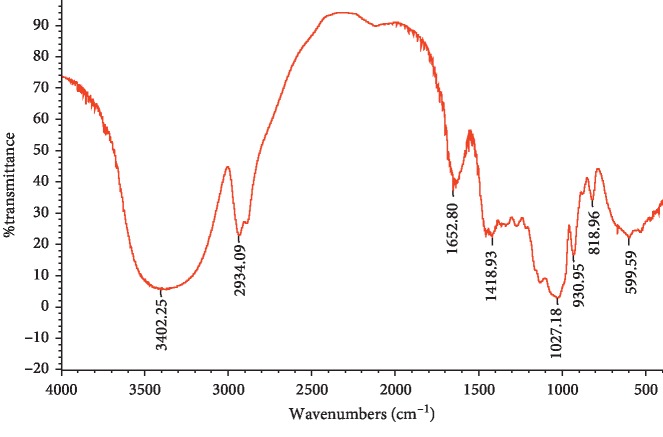
FT-IR spectra of SMP.

FT-IR is widely used by most researchers for the determination of the molecular structure of polysaccharides and the investigation of complex polymers [18]. The SMP was scanned by infrared spectroscopy and the results were shown in Figure 2. SMP showed typical absorption peaks of polysaccharides. A broad and intense peak nearby 3302 cm^−1^ was due to the O-H stretching vibration in polysaccharide [19]. The bands centered around 2934 cm^−1^ were the characteristic absorption of C-H of sugar ring [20]. It is possible that the absorption peak of 1652 cm^−1^ was the characteristic absorption of carbonyl, aldehyde, or carboxyl groups [21]. The C-H deformation vibration of the sugar ring was observed ranging from 1200 to 1400 cm^−1^ [22]. The perks ranging from 1000 to 1200 cm^−1^ were ascribed to the absorption of the pyranose ring [23]. The results indicated that SPM is a furanose.

### 3.3. Polysaccharide Content and Monosaccharides Composition of SMP

**Figure 3:**
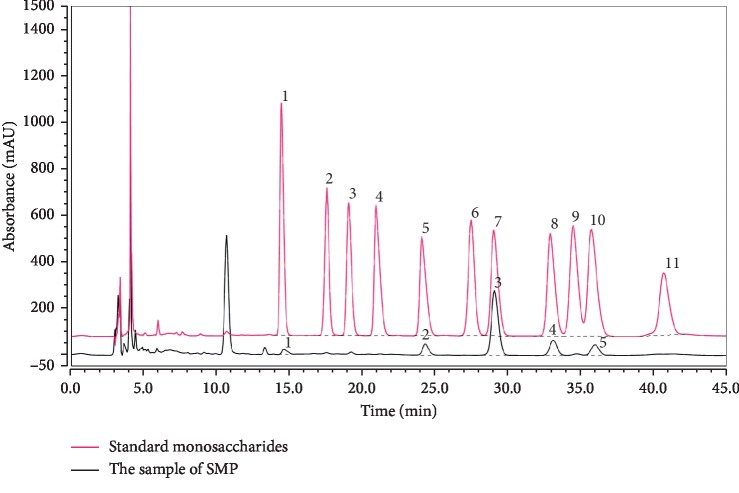
Monosaccharides compositions analysis of STP by HPLC precolumn derivatization. 1: Man, 2: GlcN, 3: Rha, 4: GlcA, 5: GalA, 6: GalN, 7: Glc, 8: Gal, 9: ara, 10: Xyl, and 11: Fuc.

The total sugar content of SMP was estimated to be 92.37%, and according to the HPLC results, SMP was composed of Glc (60.3%), Gal (15.4%), Xyl (11.1%), GalA (9.3%), and Man (3.9%) (Figure 3).

### 3.4. MICs Assay

As shown in Table 1, positive drugs have obvious inhibitory effects on specific bacteria. The MIC value of amikacin against *E. coli* was 32 *μ*g/ml and vancomycin against *S. aureus* was 16 *μ*g/ml. However, MICs of SMP against *E. coli* and *S. aureus* were both determined to be 250 mg/ml. This inhibitory concentration of SMP on *E. coli* and *S. aureus* is too high to be used in practical situations; therefore, we can conclude that SMP has no direct inhibitory effect on these two bacteria.

### 3.5. The Mortality, Enumeration of WBC, Serum IL-1, IL-6, TNF-*α*, and IgM

In this animal experiment section, we found that gender did not make any difference in all the mortality, WBC, serum IL-1, IL-6, TNF-*α*, and IgM data (data not shown).

The number of survivors and mortality data for each group were shown in Table 2. As this part of the trial focused on the effect of different treatment methods on the mortality of bacteria-infected mice, the blank control group (uninfected and without intervention) was not involved in the comparison. Crosstabs analyses showed that although it is not as significant as the effects of positive drugs (*P*=0.025 of *E. coli* infected groups and *P*=0.003 of *S. aureus* infected groups), it is easy to find that SMP can reduce the deaths of mice which was infected with *E. coli* or *S. aureus*.

The body expends a lot of energy when fighting with infection. Although SMP has no direct antibacterial activity, it belongs to the carbohydrate which can provide energy for the body. It has been confirmed that the infection of *E. coli* or *S. aureus* can cause an apparent oxidative reaction in the body [24, 25]. According to the above results, SMP can improve the survival rate of *E. coli* or *S. aureus* infected mice as SMP has certain energy providing and antioxidant activity.

**Figure 4:**
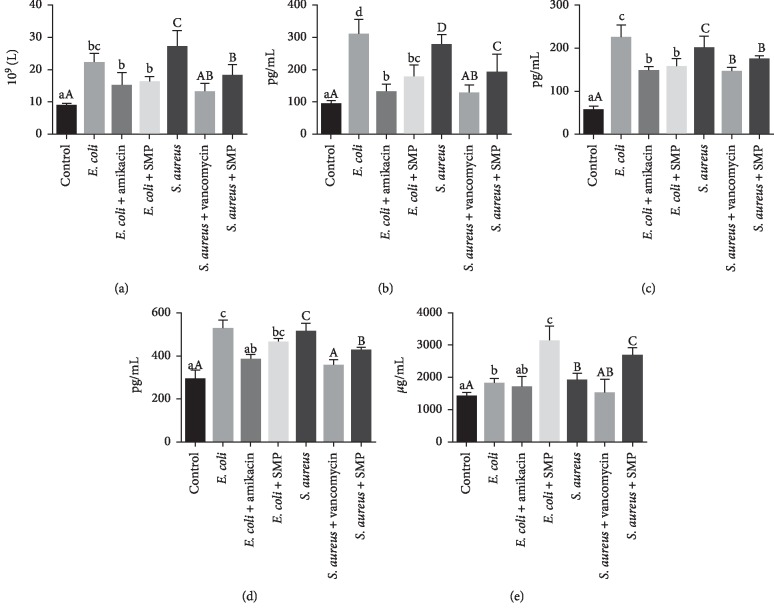
The WBC (a) and production of serum IL-1 (b), IL-6 (c), TNF-*α* (d), and IgM (e) of mice in different groups. Control: uninfected and without intervention. *E. coli* or *S. aureus*: infected with *E. coli* or *S. aureus* and without intervention. *E. coli* + amikacin or *S. aureus* + vancomycin: as positive controls, mice infected with *E. coli* or *S. aureus* were subcutaneously injected with amikacin or vancomycin at 5 hours after infection, respectively. *E. coli* + SMP or *S. aureus* + SMP: after treated with SMP for 7 days, mice were infected with *E. coli* or *S. aureus*. All the bold samples were taken on day 9. Values in a column with different superscripts (a–d, or A-D) were significantly different (*P* < 0.05).

The results of WBC count were measured by a routine blood test. According to the test report, the reference range of WBC is 0.80 to 10.60. As shown in Figure 4(a), the numbers of WBC in mice were significantly increased by the bacterial infection (*P* < 0.05). Although the data was still above the normal level, positive drugs and SMP significantly reversed these alterations induced by *E. coli* and *S. aureus* (*P* < 0.05).

The changes in IL-1, IL-6, TNF-*α*, and IgM concentrations in serum samples of each group were illustrated in Figures 4(b)–4(e). The range of references provided by the kit company is as follows: IL-1, 85.6–124.65 pg/mL; IL-6, 45.85–92.75 pg/mL; IgM, 1000.05–2106.25 *μ*g/mL; TNF-*α*: 205.5–345.75 pg/mL.

The expressions of IL-1, IL-6, and TNF-*α* in serum were significantly upregulated in bacterial infection group compared with the control (*P* < 0.05). The treatment of positive drugs decreased the levels of these expressions remarkably relative to the *E. coli* and *S. aureus* group (*P* < 0.05) and brought these indicators closer to normal values (range) than those of SMP pretreatment. Although SMP did not significantly reduce the expression of these inflammatory factors as positive drugs in all intervention groups, it can be seen that SMP has a certain anti-inflammatory ability (Figures 4(b)–4(d)).

When the mice were infected with bacteria, the IgM expressions raised compared to the control group (*P* < 0.05). After intervened with SMP, the IgM expressions were increased significantly compared to the infected and 2 positive drug groups (*P* < 0.05) (Figures 4(e)).

Bacteria enter and multiply in the body, triggering the body's inflammatory response and immune response. IL-1, IL-6, and TNF-*α* are important proinflammatory cytokines and inflammatory mediators, which are critical in the inflammation-associated and immunization-associated response [26–29]. The above cytokines are mainly secreted by white blood cells, so the concentration of cytokines that are secreted is related to the number of white blood cells. Our results also confirm this phenomenon. The WBC value of the SMP pretreatment group was significantly higher than that of the control group (*P* < 0.05) and beyond the normal range; as a result, the content of other inflammatory factors was also at a higher level. This suggests that the body is still in an inflammatory state. In this respect, positive drugs (amikacin and vancomycin) worked well because they can inhibit the pathogen directly. IgM mainly exists in the blood of organisms and is also the earliest antibody in the initial humoral immune response, which plays a vital role in the immune response. Studies have shown that the levels of IL-1, IL-6, TNF-*α*, and IgM in mice increased significantly in the acute stage of *E. coli* and *S. aureus* infection [30–33]. Our test results are consistent with those reported above. The body responds to infection by secreting a variety of substances that regulate immune and inflammatory responses, which requires a lot of energy in these processes. SMP is a polysaccharide, a type of sugar that provides energy to the body when taken in it. At the same time, SMP can reduce the level of the inflammatory response by downregulating WBC, IL-1, IL-6, and TNF-*α*; meanwhile, it can improve immune function by promoting the expression of IgM even though SMP has no direct bacteriostatic activity. Moreover, SMP has a certain antioxidant effect and it is difficult to study the whole mechanism through certain means of in vivo research. According to ancient books, *Semiaquilegia adoxoides* (DC.) Makino can cure acute infectious diseases caused by *S. aureus* and *E. coli* in clinical infection and the results of this experiment confirm this statement in some way. This trial is a preliminary study, and we plan to take further research on the biological activity of SMP in the future.

## 4. Conclusions

We concluded that polysaccharide from *Semiaquilegia adoxoides* (DC.) Makino (SMP) was a furanose and mainly composed of glucose (60.3%) and, at the same time, it has a potential antioxidant effect. SMP also had no antibacterial activity but raised the IgM and reduced WBC, IL-1, IL-6, and TNF-*α* to upregulate immunity and downregulate inflammation, leading to lower mortality of mice infected with *S. aureus* and *E. coli*. This work indicated that SMP could be explored as a promising antibacterial agent in the food and pharmaceutical industries.

## Figures and Tables

**Table 1:** The MIC values of different drugs for *E. coli* and *S. aureus*.

	*E. coli*	*S. aureus*
Amikacin (*μ*g/ml)	32	—
Vancomycin (*μ*g/ml)	—	16
SMP (mg/ml)	250	250

**Table 2:** Number of survivors and mortality data of each group.

Group	Control	*E. coli*	*E. coli* + amikacin	*E. coli* + SMP	*S. aureus*	*S. aureus* + vancomycin	*S. aureus* + SMP
Total	24	24	24	24	24	24	24
Deaths	0	13	4	9	14	3	7
Survivors	24	11	20	15	10	22	17
Mortality	0%	54.2%^a^	16.7%^b^	37.5%^ab^	58.3%^A^	12.5%^B^	29.2%^AB^

Note: line data marked without the same superscripts (a–b or A-B) differ significantly (*P* < 0.05).

## Data Availability

The data used to support the findings of this study are available from the corresponding author upon request.
